# Diagnosing challenges and setting priorities for sustainable water resource management under climate change

**DOI:** 10.1038/s41598-022-04766-2

**Published:** 2022-01-17

**Authors:** Ibrahim Nourein Mohammed, John D. Bolten, Nicholas J. Souter, Kashif Shaad, Derek Vollmer

**Affiliations:** 1 Science Applications International Corporation, Hydrological Sciences Laboratory, NASA Goddard Space Flight Center, Mail Code 617.0, Greenbelt, MD 20771 USA; 2 Hydrological Sciences Laboratory, NASA Goddard Space Flight Center, Mail Code 617.0, Greenbelt, MD 20771 USA; 3 Conservation International, Greater Mekong Program, Adelaide, SA 5005 Australia; 4 Conservation International, Betty and Gordon Moore Center for Science, Arlington, VA 22202 USA

**Keywords:** Hydrology, Climate-change adaptation, Climate-change impacts, Climate-change policy, Environmental impact, Sustainability, Hydrology, Climate and Earth system modelling, Climate-change impacts, Projection and prediction

## Abstract

Managing transboundary river basins requires balancing tradeoffs of sustainable water use and coping with climate uncertainty. We demonstrate an integrated approach to exploring these issues through the lens of a social-ecological system, combining remote and in-situ earth observations, hydrologic and climate models, and social surveys. Specifically, we examine how climate change and dam development could impact the Se Kong, Se San and Sre Pok rivers in the Mekong region. We find that climate change will lead to increased precipitation, necessitating a shift in dam operations, from maintaining low flows to reducing flood hazards. We also find that existing water governance systems in Laos, Vietnam, and Cambodia are ill-prepared to address the problem. We conclude that the solution space for addressing these complex issues will be highly constrained unless major deficiencies in transboundary water governance, strategic planning, financial capacity, information sharing, and law enforcement are remedied in the next decades.

## Introduction

Sustainable water resource management is fraught with uncertainties and indeterminate scope, particularly in transboundary river basins that may include divergent social values and stakeholder interests^1^, and hydroclimatology which is in constant flux^2,3^. Water management decisions take place at many scales, but it is often at the river or lake basin scale where tradeoffs must be assessed—among jurisdictions demanding water, among different economic uses for that water, and between human and ecological needs^4^. Climate change is adding more uncertainty and, in many places, will amplify challenges by exacerbating extreme hydrologic events^5^. It is clear that decision makers will need to evaluate tradeoffs across sectors (e.g., hydropower versus fisheries), beneficiaries (upstream versus downstream), and generations, since hydropower dams and climate change induce long-term, largely irreversible alterations to water systems^4,6–11^.

A logical response to these pending issues has been to develop and quantitatively model future scenarios that help identify specific challenges and the solution space for water resource managers. But typically, these analyses overlook the water governance system in place, which determines what is a feasible course of action for planning and mitigation^12–14^. It is against this backdrop that we developed this study, recognizing that *problem definition* is critical in water resource management studies, and that institutional context is a core part of this^2,15^.

Here, we present an example of an integrated approach to assessing future sustainability challenges in their social, hydrological, and ecological dimensions using a case study from the Lower Mekong basin. Climate change could have a more substantial impact on hydropower here than elsewhere in Asia^16^ but could also lead to declining rice yields^17^, lower sediment delivery^18^ and greater salinity intrusion in the delta^19,20^. Our study area is the combined basin of the Se Kong, Se San and Sre Pok (3S) rivers, which deliver approximately 20% of flow^21^ and 25% of total sediment load^22^ to the Mekong River system (Supplementary Information—Figure S1). The Se Kong River originates in Lao PDR and the Sre Pok and Se San rivers rise in the central highlands of Vietnam; all three rivers merge in Cambodia shortly before flowing into the main stem of the Mekong River. The 3S River Basin supports a population of approximately 3.4 million with low levels of socio-economic development and population centers in close proximity to the rivers and their tributaries. Extensive hydropower development has altered the flow regime, sediment transport, and fish migration with broader implications for the Lower Mekong Basin including the sustainability of the Tonle Sap Lake and the Mekong delta.

### Assessing a realistic “solution space” for sustainable water management

Recent studies of the 3S River Basin have employed hydrologic and other numeric models to evaluate potential tradeoffs^23^, providing insights into dominant drivers of hydrologic alteration^20^ or various sources of uncertainty^24,25^. But translating quantitative modeling results into decision-relevant information also requires an improved understanding of the social dynamics of a water system^26,27^. Studies recommending integrated operation of dam cascades^28^ or coordinated regional development of dam siting^29^, for example, have not considered the governance systems in place and the very real constraints they place on any solution set. These challenges are magnified in rapidly developing transboundary basins, where water resources are strongly influenced by national decisions on land use and infrastructure, regional geopolitical considerations, and the willingness and ability of basin countries to cooperate^1^.

This study uses a mixed methods approach to analyze potential impacts of climate change on regional hydrology, the ability of dam operation rules to keep downstream flow within acceptable limits, and the present state of water governance in each country. To define the solution space with regards to climate change and water resource tradeoffs, we use a calibrated hydrologic model leveraging satellite-based remote sensing for the Lower Mekong basin^30^ and the NASA Earth Exchange Global Daily Downscaled Projections (NEX-GDDP)^31^ for scenarios of changing climate. To interpret these modeled results, we use several indicators from the Freshwater Health Index^32^ and its social-ecological system framework to evaluate predicted impacts to the *natural* (i.e., pre-development period) flow regime and flood regulation. From the results of a perception-based Governance and Stakeholders survey completed by a select group of regional decision makers^33^ and international subject matter experts we examined seven indicators: Strategic Planning and Adaptive Management, Water Resource Management, Distribution of Benefits from Ecosystem Services, Water Related Conflicts, Enforcement, Information Access and Knowledge, and Financial Capacity in detail.

## Results

For the 3S River Basin, we modelled twenty-four scenarios examining the interaction between a suite of climate projections and varying operational rules for 23 dams (i.e., 21 currently operating, and 2 planned/under construction) to capture a range of climate- and human-caused factors that influence streamflow dynamics and management. We find that predicted climate change will lead to more precipitation, increased seasonal streamflow variability (e.g., larger flood peaks) and that dam operation will have limited ability to adapt to the changing flow regime. The predicted increase of seasonal streamflow variability has multiple layers of uncertainty that are related to observational data, the nature of the physical modeling conducted, and the implemented climate change models data (e.g., aerosol radiative forcing of climate). Overall, river flows could move closer to *natural* conditions, but the likelihood of floods will increase, creating a new management objective for dam operations. These impacts vary among the three rivers, signaling a need for strategies tailored to the individual sub-basins, as well as highlighting the need for greater coordination between upstream (Laos and Vietnam) and downstream (Cambodia) countries.

We examined four global climate model groups^34^ and two representative CO_2_ concentration scenarios^35^ (i.e., RCP45 and RCP85) under the Intercomparison Project Phase 5 (CMIP5)^36^. Namely, these four climate modeling groups are: the National Center for Atmospheric Research, NCAR (CCSM4); the NOAA Geophysical Fluid Dynamics Laboratory, NOAA GFDL (GFDL—CM3); the Institut Pierre-Simon Laplace (IPSL—CM5A—MR); and the Norwegian Climate Centre (NorESM1—M). Table S1 gives the selected global climate groups to conduct this work. The climate model groups examined varied from dry projection (GFDL—CM3) to wet projection (NorESM1—M). Figure S3 (Supplementary Information) depicts the Lower Mekong River Basin climate projection.

The climate datasets were compared against three reservoir release rule scenarios: (a) *Business as Usual* (BAU), which follows the current Vietnamese dam operation rules obtained from the Vietnamese National Mekong Commission (b) *Storage*, which is a 50% reduction in dry season/minimum release targets, and (c) *Release*, which is a 100% increase in dry season/minimum release targets. Details for management scenarios examined are provided in supplementary information Table S2. While these scenarios are simplistic and applied uniformly to all dams, they provide a useful envelope for estimating the range of potential impacts from dramatically changing dam operation rules. Leveraging an established methodology that isolates and scores the ecosystem risks and benefits of changing water landscapes^33,37,38^, we then used the social-ecological framework of the Freshwater Health Index^32^ to compare the results of these scenarios and their relative impacts on key indicators of ecological health and human well-being.

### Returning to a more natural flow regime in the dry season

The suite of climate models predicts an increase in annual precipitation of around 6 mm/year from 2025 to 2050 over the Lower Mekong region (Figure S3—Supplementary Information). The period of rainfall during the wet season will likely be shorter, but more intense. Annual maximum air temperature is projected to increase over the Lower Mekong by 2.7 °C [1.6 °C, 3.8 °C] (± 95% confidence interval)—275.85 K [274.75 K, 276.95 K]. We examined the temporal and spatial aspects of the future flow-regime caused by the combined effects of predicted climate change and human impacts through dam operations.

**Figure 1:**
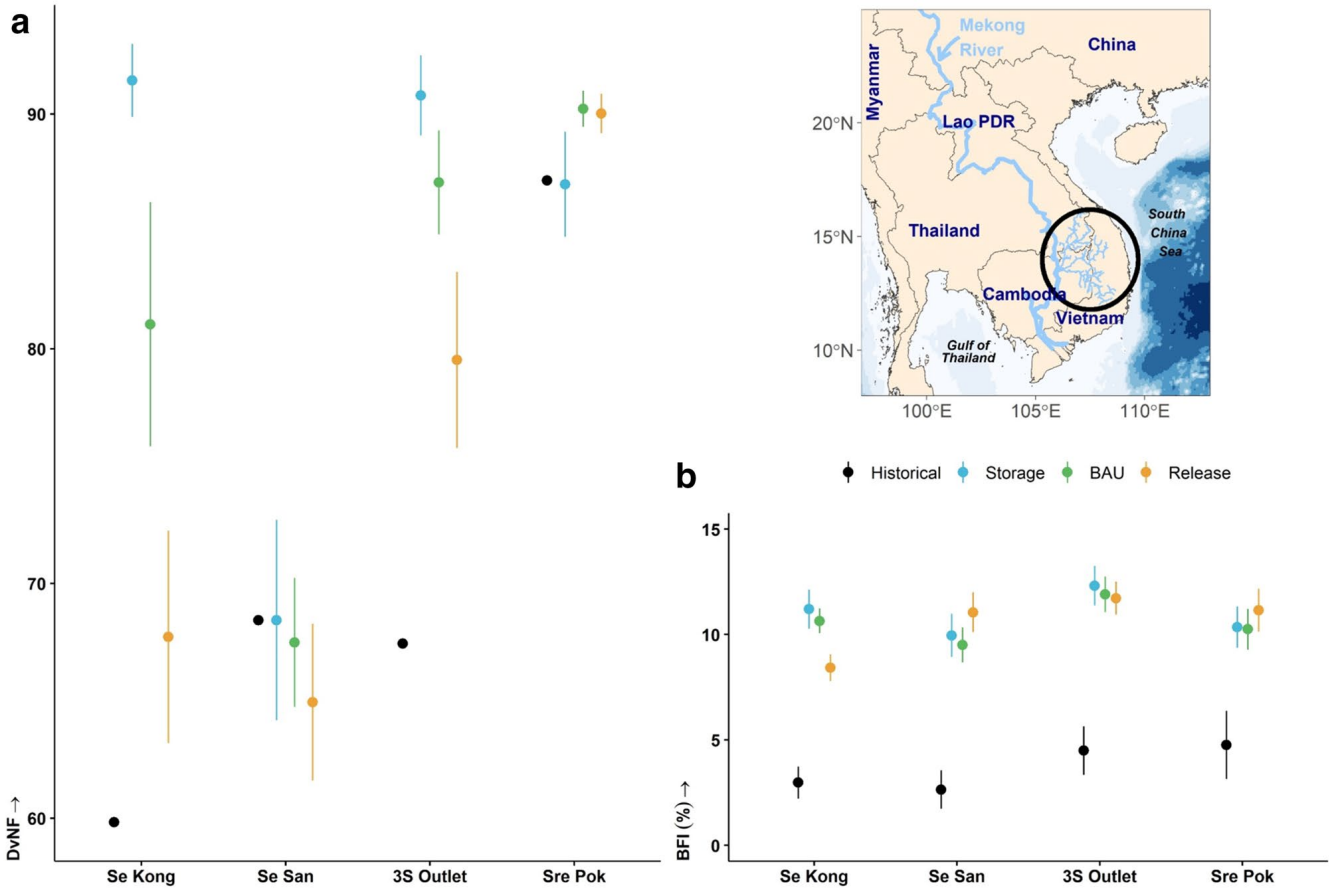
Se Kong, Se San and Sre Pok (3S) River Basin flow regime under 24 climate change and management scenarios (**a**) mean (± 95% CI) deviation from natural flow (DvNF) (**b**) mean (± 95% CI) base flow index (BFI). Historical results calculated from the 2002—2018 time period, climate and management scenarios calculated from 2025—2050 time period. Map created and drafted using R: A language and environment for statistical computing version 4.0.3: https://www.R-project.org/ (Vienna, Austria). The map layout was plotted using EPSG Geodetic Parameter Dataset 4326 projection (https://epsg.io/4326).

We estimated the Deviation from Natural Flow $\left( {DvNF} \right)$^32,39^ metric at 177 river reaches above and below the 23 current and planned reservoirs following the three sets of dry season management rules. The 3S River Basin reaches network was extracted from the digital elevation model (DEM). With the largest estimated difference in $DvNF$, the Se Kong River was most sensitive to management rules. And with the anticipated climate-induced increased precipitation, the current lower-than-natural flows in the dry season (due to priority for storage) will likely be reversed. Thus, at least by one measure, the three rivers may return to a flow regime under climate change that is closer to *natural* (Fig. 1a). It is important to note here that storage capacity of a reservoir influences the realized flow regime during any scenario, thus, the smaller storage capacity of the reservoirs on the Sre Pok River (1,241 Mm^3^) compared to the other two rivers allow it to maintain a flow regime closer to historic conditions across all three scenarios examined. The modelled storage capacity for the Se Kong River and the Se San River were 9,842 Mm^3^ and 5,128 Mm^3^ respectively. Moreover, it is likely that the Se San River future flow regime would maintain a $DvNF$ score similar to the historical (i.e., reservoir development in 2018) score irrespective of all the management rules being examined. That’s because many of the reservoirs on the Se San River (i.e., Lower Se San 2, Yali, and Se San 4) have current dry season discharge rules (BAU) in favor of power generation (i.e., very high flow discharge during dry season). The Se Kong River, the Se San River, and the Sre Pok River $DvNF$ scores (Fig. 1a) were calculated at river reaches crossing international borders (i.e., the Vietnam and Cambodia border for the Se San & Sre Pok Rivers, and Lao and Cambodia border for the Se Kong River). Historical flow simulation results calculated during the 2002–2018 time period were obtained from earlier model runs utilizing satellite earth observations data products^40^.

**Figure 2:**
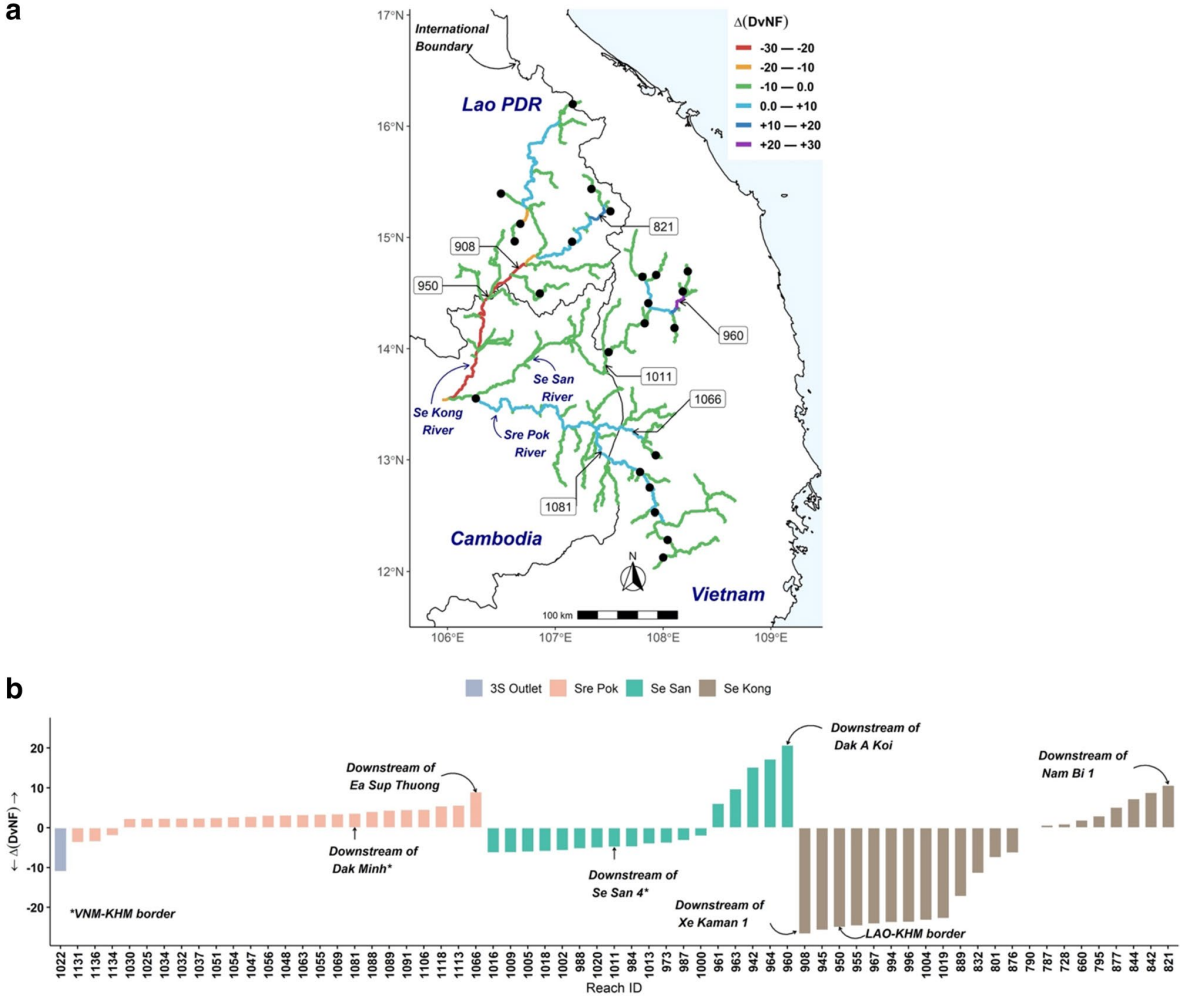
Change in deviation from natural flow ($\Delta DvNF = DvNF_{Storage} - DvNF_{Release}$) within the Se Kong, Se San, and Sre Pok (3S) River Basin under the Coupled Model Intercomparison Project Phase 5 (CMIP5) representative concentration scenario (RCP 8.5) with the GFDL—CM3 climate group in response to different management scenarios, (**a**) spatial variation of the change in deviation from natural flow, and (**b**) bar plot of the change in deviation from natural flow. Black dots are modelled existing and planned reservoirs. A zero in $\Delta DvNF$ refers to 3S River segments that are insensitive to management scenarios. The DvNF results shown were calculated from 2025 to 2050 time period. The 3S River segments are labeled with Reach ID numbers (e.g., Reach ID # 1022 is the 3S Outlet). Map created and drafted using R: A language and environment for statistical computing version 4.0.3: https://www.R-project.org/ (Vienna, Austria). The map layout was plotted using EPSG Geodetic Parameter Dataset 4326 projection (https://epsg.io/4326).

Our $DvNF$ scores for the 3S Rivers under the different management scenarios and climate models are presented in more detail in the Supplementary Information (Figure S5). Isolating the climate change impacts from the management rule impacts on the flow regime has been obtained with the $\Delta DvNF$ scores (Fig. 2a). The $\Delta DvNF$ score is calculated at each stream reach using the *Storage* and *Release* reservoir management rules $\left( {\Delta DvNF = DvNF_{Storage} - DvNF_{Release} } \right)$*.* The spatial variability of the $DvNF$ scores suggests that under the various climate change scenarios, about 37% of the 3S’s River reaches are responsive to reservoir management rules (Fig. 2b). The threshold being implied here to determine whether a stream reach $DvNF$ score is responding to a change from reservoir management rules or not is when $\left| {\Delta DvNF} \right| \succ 0$. We examined these selected 3S’s River reaches (i.e., 37% of the 3S River reaches) to examine the impact of climate change on flow regime under the various management rules discussed. Our results suggest that flow downstream of the Xe Kaman 1 at the Se Kong River has higher variability of $DvNF$ under the anticipated climate change. Overall, it can be seen that the three management scenarios can lead to a variation of about 10% in the 3S River Basin flow regime.

We calculated the base flow index^41^ (BFI) for the outlets of the Se Kong, Se San, and Sre Pok (3S) River Basin to quantify flow stability and susceptibility to extreme low flow. Based on historical streamflow data the mean of the BFI at the 3S River Basin outlet (Fig. 1b) was about 4%. Low flows were predicted to increase over the next 25 years of dry seasons to more than double the historical value. The predicted changes in low flows explain our earlier results related to the basin flow regime moving closer to the *natural*. Low flow disturbance, as reflected by BFI scores, may affect fish assemblages. And the 3S River Basin is an important component of the larger Mekong fishery^6^.

Though climate change is predicted to be a major driver of increased low flows (and a more natural regime) in our models, the magnitude of the changes in streamflow dynamics can be influenced by dam operations on the Se Kong River (Fig. 1). This illustrates the delicate balance between water governance and climate impacts on the water landscape that decision makers and managers need to consider achieving optimal water resource management. For example, our predicted shift in low flow regime will require adjustments in planning to reflect and respond to the ensuing climate-driven changes in the basin flow regime as it is anticipated to affect stream habitat and fish composition^42^.

### Increasing wet season flood risk

**Figure 3:**
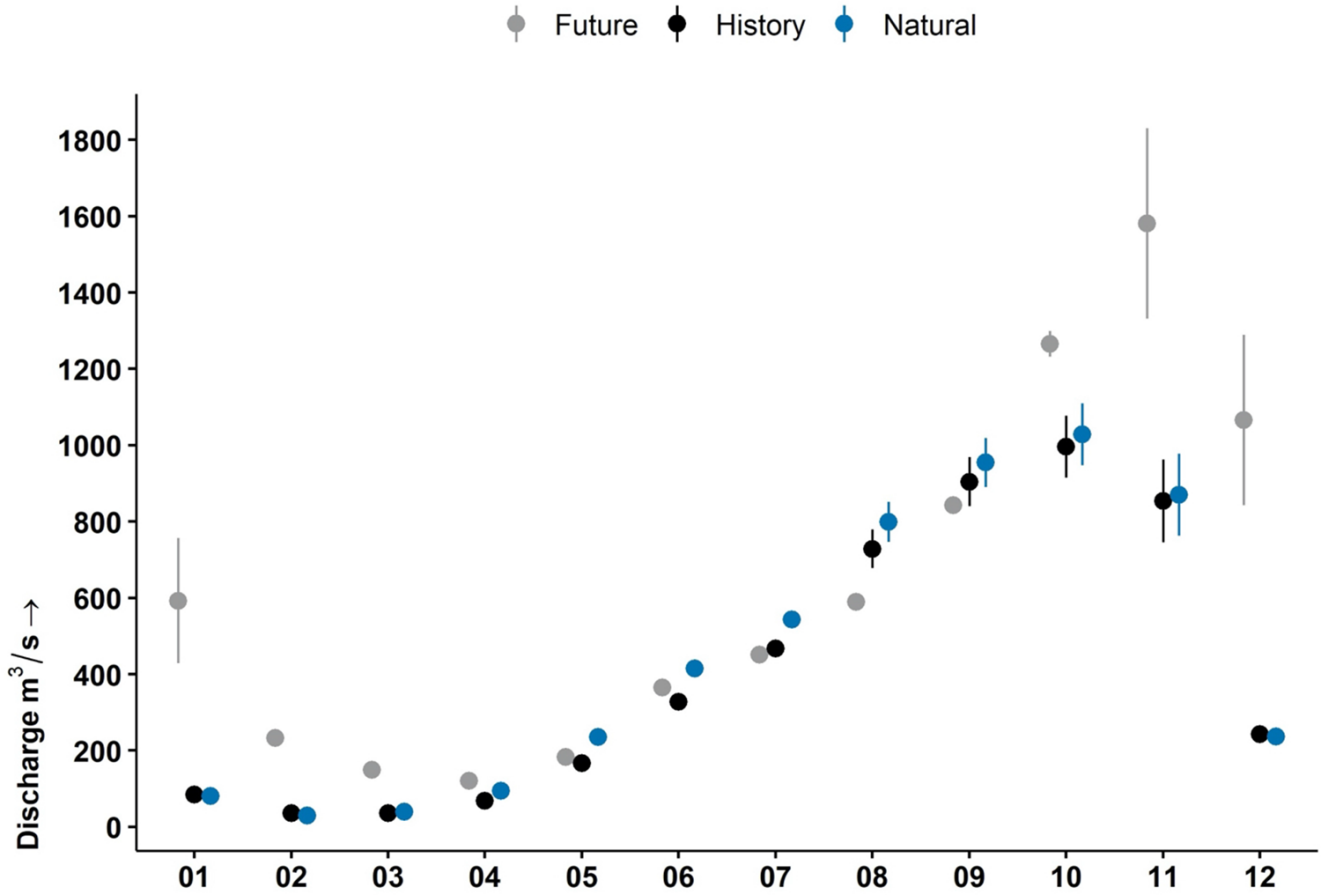
Yali Reservoir downstream flow hydrograph of mean (± 95% confidence interval) discharge under natural, historical, and future (*Business as Usual* reservoir rules) scenarios. Natural and historic discharge derived from 2002 to 2016. Future flows were calculated from four climate model groups and two greenhouse gas emissions scenarios under the Coupled Model Intercomparison Project Phase 5 (CMIP5) from 2025 to 2050.

Dams in the 3S River Basin were, and continue to be, built and operated to generate hydroelectricity, not to reduce downstream flood risk. So, the expected increase in wet season precipitation and streamflow will present a new challenge for dam operators. For Vietnam’s second largest dam, Yali Falls, which has been linked to several floods downstream in Cambodia^43^, the impacts of climate change are predicted to substantially increase discharge from October to April, peaking one month later than historically and at levels > 50% over baseline conditions (Fig. 3). A shift to a shorter and wetter dry season precipitation pattern adds new implications and challenges to the existing water management system. Broadly, our results are in agreement with a collection of studies on the changes in Mekong River flow, summarized as streamflow increases year-round^44^.

**Figure 4:**
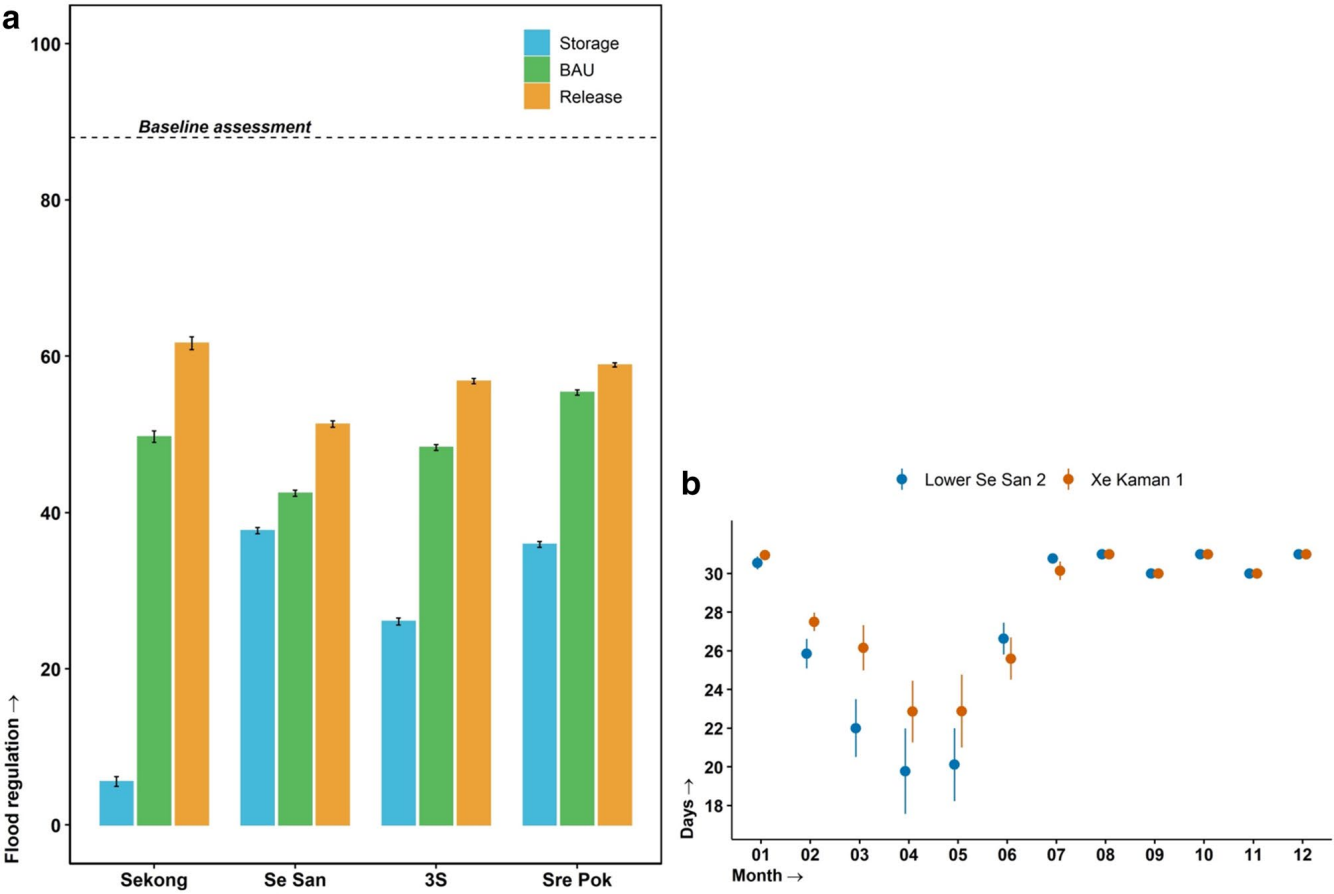
Se Kong, Se San and Sre Pok (3S) River Basin flood regime under 24 climate change and management scenarios from 2025 to 2050 (**a**) mean (± 95% CI) flood regulation (how many and how often reservoirs reach a flood storage threshold) capacity for the three management scenarios on a scale of 0 (low) to 100 (high) for each tributary and the whole 3S Basin. Baseline assessment from Souter et al.^33^. (**b**) Mean (± 95% CI) number of days with storage equal to or greater than 95% of the maximum reservoir storage volume at the Lower Se San 2 and Xe Kaman 1 dams for the *Business as Usual* management rules.

We calculated a flood regulation indicator to quantify the increased risk of flooding under the future scenarios (Fig. 4a). The flood regulation indicator assesses two dimensions of flood risk, scope, and frequency, across all the reservoirs simulated in this study. A reservoir is considered to be flooding when its storage volume equals or exceeds 95% of the maximum reservoir storage volume (Tables 1 & S2). Using this threshold, the number of reservoirs flooding (scope), and number of times each reservoir floods within the study period (frequency) is calculated and mapped on a scale of 0 to 100—where 0 indicates low, and 100 high, capacity for flood regulation. Our results suggest that the 3S River Basin system is expected to experience new patterns and amounts of precipitation that could contribute to more frequent floods. The baseline assessment^33^ (88 out of 100, highlighted in Fig. 4a), which was derived from the frequency and amplitude of monitored flow exceeding the flood thresholds of four gauging stations within the 3S River Basin, whilst not directly comparable with the method used in our current assessment, does show that flooding is currently well managed within the system.

**Table 1:** The ecological and social framework used in examining the climate change and dam development impacts on the sustainability of the Se Kong, Se San and Sre Pok (3S) rivers in the lower Mekong River basin.

Major indicator	Sub-indicator	Metric	Site & scale datasets	Notes
**Ecosystem vitality**				
Water Quantity	Deviation from Natural Flow Regime	AAPFD & DVNF	River reaches	Gehrke et al.^39^
Flow Stability	Base Flow Index	BFI	River reaches	Poff^41^
**Ecosystem services**				
Regulation & Support	Flood regulation	Aggregate of sites affected, frequency and amplitude of floods	Dams	Flood threshold is reservoir volume storage equal to or exceeding 95% of the maximum reservoir storage volume
**Governance & Stakeholders**				
Enabling Environment	Water Resource Management Financial Capacity	Questionnaire survey	Regional expert input	Vollmer et al.^32^ & Souter et al.^33^
Stakeholder Engagement	Information Access and Knowledge			
Effectiveness	Enforcement and Compliance Distribution of Benefits from Ecosystem Services Water-related conflict			
Vision and Adaptive Governance	Strategic Planning and Adaptive Governance			

For each of the three rivers, and the system as a whole, the storage scenario had the lowest flood regulation scores, all of which were half the baseline score (Fig. 4a). We expected the storage scenario to reduce flooding and flood damage by slowing peak flows, however the low scores suggest that this management regime would not be able to cope with the predicted repeated high inflows. We attribute these poor flood regulation scores to slow releases of reservoir water storages and the long residence times. Also, our results may require a revision of existing management rules (BAU) since flood regulation scores for all rivers and the 3S River Basin as a whole were below 60 (except Se Kong River with *Release* management rules), a point at which the ecosystem service is not being adequately met^33^. However, releasing more water from reservoirs in the dry season to reduce reservoir water volumes in anticipation for wet season inputs will not help to absorb the expected high pulses of water during wet seasons. These findings necessitate new flood regulation policies in all three rivers and the whole basin with specific attention paid to setting minimum reservoir storage capacity volumes to decrease peak flows amplitude.

We calculated the projected (i.e., 2025–2050) seasonal number of days when the reservoir volume storage is equal to or greater than 95% of the maximum reservoir storage volume (i.e., emergency spillway volume) at two different reservoirs under the BAU management scenario (Fig. 4b). This highlights the near constant need to manage flood waters in reservoirs further down the cascade. Whilst we assessed these changes through the lens of flow dynamics and flow regime, we did not examine many other attributes related to the anticipated environmental conditions as a result of the expected change in flow regime. A coordinated and enforced management plan between the 3S River Basin’s riparian countries will be needed to manage future floods and remediate their impact.

### Deficiencies in water governance

**Figure 5:**
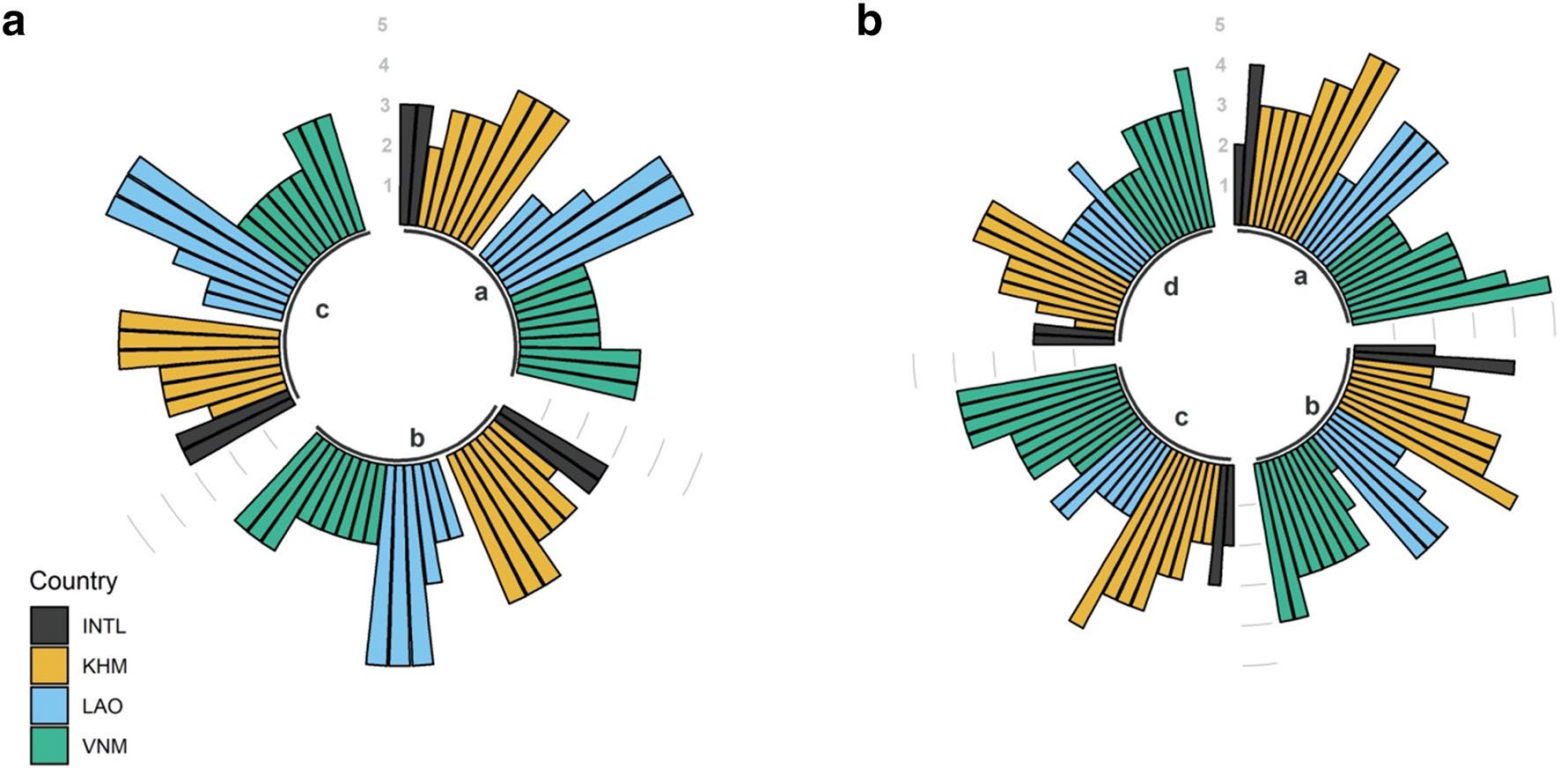
Governance and Stakeholders survey responses for (**a**) Strategic planning and adaptive management (a, shared vision; b, strategic planning mechanisms; and c, adaptive management framework.); and (**b**) Water Resource Management (**a**, coordinated policies and actions; **b** centrally managed infrastructure; **c** financial resources; and **d** ecosystems conservation priorities). Full descriptions of each survey response and scale categories are provided in the Supplementary Information. Response country codes are: INTL (International), KHM (Cambodia), LAO (Laos, PDR), and VNM (Vietnam).

Against this predicted future of increased river flow, sufficient to shift reservoir operational priorities, is a backdrop of underdeveloped water governance and limited stakeholder engagement^33,45^. The overall Governance & Stakeholders survey assessment gave a score of 43 (out of 100) with all indicators scoring poorly^33^ (Tables 1, 2, Fig. 5, and Figures S6 to S10). Strategic planning and adaptive management are vital to managing the 3S’s changing future. But, with an indicator score of 47 there is limited capacity to achieve this, and a score of 34 from the Vietnamese respondents is concerning, as the majority of the 3S’s dams are in Vietnam (Fig. 5a). The majority of Vietnamese respondents rated the various processes for strategic planning and adaptive management as “rarely comprehensive” (2 out of 5), whilst “sometimes comprehensive” (3 out of 5) was the highest score; this is consistent with a study of readiness for adaptive freshwater management in the Vietnamese Mekong Delta^46^. Responses from Laos and Cambodia were more variable but, on average, still low, casting further doubt on decision makers’ collective ability to implement effective strategic planning and adaptive management.

**Table 2:** Governance survey description and indicator questions for the Se Kong, Se San and Sre Pok (3S) Rivers’ stakeholders.

Major indicator	Sub-indicator	Description	Indicator Questions	Likert Scale Key
Enabling Environment	Water Resources Management	Integrated water resources management is a guiding framework for coordinating both development and management of all resources within a basin, to maximize welfare without compromising ecological sustainability. In some cases, a single agency, such as a river basin authority, is responsible for coordinating and overseeing these functions; the questions below focus on the specific functions as managed within your jurisdiction (e.g., transnational, national or provincial) regardless of whether they are all carried out by the same agency	(a) Policies and actions to advance water resource development and management are coordinated	1. Function is almost never satisfactory (without conflicts among stakeholder groups)
			(b) Infrastructure such as dams, reservoirs, and treatment plants are centrally managed or coordinated	2. Function is rarely satisfactory
			(c) Financial resources are mobilized to support water resource development and management needs	3. Function is sometimes (~ 50%) satisfactory
			(d) Ecosystems conservation priorities are developed and actions implemented	4. Function is often satisfactory
				5. Function is almost always satisfactory
	Financial Capacity	Water resource development and management is often under-financed, particularly for services that do not generate revenue, such as ecosystem protection. Although financial capacity can be measured directly as a function of existing allocations relative to estimated budget needs, qualitative information is also useful in providing insights and identifying priorities	(a) Level of investment in water supply development	1. Level is very unsatisfactory
			(b) Level of investment in service delivery systems	2. Level is unsatisfactory
			(c) Level of investment in wastewater handling and treatment	3. Level is satisfactory
			(d) Level of investment in ecosystem conservation and rehabilitation	4. Level is very satisfactory
			(e) level of investment in monitoring and enforcement	5. Level is extremely satisfactory
Stakeholder Engagement	Information Access and Knowledge	Sound water governance requires information on a range of topics and from many sources. Even in cases where data and information are abundant, if they are not made accessible (across agencies, with citizens, etc.) then they are less likely to aid in wise decision making	(a) Information is accessible to interested stakeholders	1. Level is very unsatisfactory
			(b) Information meets expected quality standards, in terms of frequency, level of detail, and subjects of interest to stakeholders	2. Level is unsatisfactory
			(c) Information is transparently sourced	3. Level is satisfactory
			(d) All available, sound and relevant information is routinely applied in decision-making	4. Level is very satisfactory
				5. Level is extremely satisfactory
Effectiveness	Enforcement and Compliance	In many societies, there is a gap between laws and their actual enforcement, reflecting either insufficient capacity or a lack of accountability. Enforcement and compliance can be ensured through fines, incentives, or social pressure, but weak enforcement leads to poor management and a lack of confidence in the system	(a) Surface water abstraction guidelines are enforced	1. Enforcement is very poor or no guidelines (formal or informal) exist
			(b) Groundwater abstraction guidelines are enforced	2. Enforcement is poor
			(c) Flow requirement guidelines are enforced	3. Enforcement is acceptable
			(d) Water quality guidelines are enforced	4. Enforcement is good
			(e) Land use guidelines are enforced	5. Enforcement is very good
	Distribution of Benefits from Ecosystem Services	Equity is an important issue in water resource management, most closely associated with access to safe water and sanitation. Here we extend the concept to include all benefits from ecosystem services in the basin (water and sanitation, fisheries, flood mitigation, water quality maintenance, disease regulation, and cultural services)	(a) Economically vulnerable populations benefit from ecosystem services	1. Their share of benefits is almost never adequate
			(b) Indigenous people benefit from ecosystem services	2. Their share of benefits is rarely adequate
			(c) Women and girls benefit from ecosystem services	3. Their share of benefits is sometimes (~ 50%) adequate
			(d) Resource-dependent communities benefit from ecosystem services	4. Their share of benefits is often adequate
				5. Their share of benefits is almost always adequate
	Water-related conflict	Tensions among stakeholders are expected when there is competition for scarce resources such as water. An effective governance system should prevent tensions from escalating into conflicts, here defined as a difference that prevents agreement, and therefore delays or undermines a decision taken with the basin	(a) Frequency of conflict due to overlapping jurisdictions (e.g., between national governments in transboundary systems, provincial and national government, or between agencies)	1. Conflicts almost always occur
			(b) Frequency of conflict about water rights allocation	2. Conflicts often occur
			(c) Frequency of conflict about access	3. Conflicts sometimes occur
			d) Frequency of conflict regarding the siting of infrastructure	4. Conflicts rarely occur
			(e) Frequency of conflict over water quality and other downstream negative impacts	5. Conflicts almost never occur
Vision and Adaptive Governance	Strategic Planning and Adaptive Governance	Comprehensive planning is the process of developing goals and objectives concerning water quantity and quality, surface and groundwater use, land use change, river basin ecology, and multiple stakeholders’ needs. Adaptive management refers to the ability to handle changes, unintended consequences, or surprises to the water resource system through updating planning and processes using new information	(a) A shared vision is established and used to set objectives and guide future development	1. Process is almost never comprehensive, or does not occur at all
			(b) The existence and use of strategic planning mechanisms	2. Process is rarely comprehensive
			(c) The existence and use of an adaptive management framework	3. Process is sometimes (~ 50%) comprehensive
				4. Process is often comprehensive
				5. Process is almost always comprehensive

Further complicating effective strategic planning is the need for transboundary cooperation. The poor score for Water Resource Management (50) suggests that this indicator’s varied components were only sometimes satisfactory. Managing the predicted increase in flow and it’s variability, as revealed by the Deviation in Natural Flow results above, between sub-basins and river sections will require a high degree of central coordination in infrastructure such as dams and reservoirs. Here again, the results from Vietnam are concerning, as whilst most respondents rated coordinated management as sometimes satisfactory, responses ranged from often to almost never satisfactory (Fig. 5b). This widespread disagreement may be indicative of different perceptions among stakeholders as to how the system should be managed. This notion is supported by the results of both the distribution of benefits from ecosystem services (42) and water related conflicts (45) indicators, where there was considerable variability in responses within and between the three countries.

Implementing integrated trans-boundary management will also be hampered by practical considerations such as the low level of financial capacity (36), limited information access and knowledge (41), and weak enforcement and compliance (37) in the basin. The majority of respondents rated both investments in monitoring and access to information as unsatisfactory. Thus, managing the downstream impacts of future overflow dam releases will require a significant improvement in information gathering and communications to avoid flood damage. The financial resources needed to support water resources development and management needs also scored poorly, highlighting the need for greater investment and cost-recovery in water resource management in all three countries. The current poor levels of enforcement and compliance with existing water laws would undermine efforts to transform the governance regime. Much has already been written about the challenges of water governance in the Mekong region, but this analysis provides the first self-assessment by regional decision makers, confirming that several key indicators are severely lagging.

## Discussion

Sustainably managing resources in a transboundary freshwater basin is a complex problem, particularly when considering the compounding impacts of climate change, hydropower development, and evolving water governance paradigms. We approached this problem through the social-ecological lens of freshwater health^32^, incorporating facets of the physical and social aspects of water management to explore tradeoffs as well as the limits imposed by the current governance system. This reinforces the fact that the solution space is confined by decision makers’ ability to gather information, develop, and implement plans based on that information, and adapt to changing conditions. We consider this assessment of the governance system as a critical step in evaluating hydrologic change and potential management responses, and one that is often absent in modeling studies, which can lead to proposing solutions ill-fitted to their context.

Our results indicate that the solution space needs to consider the predicted climate induced impacts on water resources in the 3S River basin—while this is not surprising, it is not yet common practice in the region, and our approach of using widely available data and a limited set of indicators can be a starting point. We have attempted to segregate the climate change impacts from the management impacts on flow regime at our study area to better understand the limits of changing dam operation. We think that these results could help guide future reservoir operational policies, where there will likely be a need to shift fairly dramatically towards flood mitigation in the wet season. In this context, transparency and cooperation (across sectors and jurisdictions) are not aspirational—they are foundational to the three countries’ ability to adapt to a changing flow regime. We focused on dam operation but there are several alternatives to mitigate flood risk, from early warning systems to green infrastructure solutions like reclaiming floodplains and restoring headwater forests. The potential impact of these solutions can be incorporated into our modeling framework, and in many instances might be preferable to conventional hard infrastructure solutions, but would still be constrained by the countries’ ability to implement and manage them^47^.

The predicted climate induced increase in reservoir overflows could have major impacts on the structural integrity of the basins’ dams^49^. High flows will see more water being discharged over spillways and into stilling basins, both of which may need expensive upgrades to remain safe. Hydropower dams in Laos and Cambodia are largely financed under Build, Operate, Own, and Transfer (BOOT) contracts, where a private sector company builds and operates the dam for a fixed period before handing it over to the government. For example, the Lower Se San II dam was built under a 45-year BOOT contract^50^. Hydropower financing in the region involves opaque processes and confidential documents^51^ and it is therefore unclear who will take responsibility for climate induced infrastructure upgrades in the second half of the dam builders’ ownership concession. Thus, it is a risk that these dams will prove to be a dangerous burden on the Governments of Laos and Cambodia who, at least for now, lack the financial capacity to mitigate potential structural problems. Future hydrologic change in the 3S River Basin is also going to alter sediment transport downstream into the Tonle Sap Lake and Mekong delta. We did not factor sediment-induced reservoir capacity reduction in our modeling, but this provides another argument for facilitating more sediment passing through them to maintain reservoir capacity and support downstream ecology. This of course has financial implications as well, as retrofits can be extremely costly, if they are even possible^22^.

Remote sensing and modeling, as we have demonstrated, can contribute to filling information gaps and offer a comprehensive view of the basin, in particular, to help understand the nature and amount of change in flow regime under climate change scenarios. We identified opportunities to focus on managing a river or individual reaches to minimize negative impacts, but this approach cannot be prescriptive—riparian countries first need to agree on the severity of impacts and their respective rights and responsibilities regarding shared waters^48^. Water governance, particularly in transboundary systems such as the 3S River Basin, is often the source of water crises^45^. Here, systems not facing imminent threats or chronic water shortages are nonetheless vulnerable to water insecurity if the water governance system is underdeveloped or underperforming. This is an indication that decision makers are ill prepared to navigate challenges arising from further hydrologic alteration in the basin, whether from development projects or climate change. In this case, our assessment reveals that the basic building blocks of good water governance, such as financing, information sharing, and enforcement, require substantially more attention in the coming years. It will be of little use to search for optimal solutions that are not fit for the context, or to invest in costly infrastructure if there is not a similar commitment to strengthening water governance and management in the region.

## Methods

To undertake this assessment, we strategically combined Mohammed et al.’s^40^ water resources modeling and tools^52^, with the Freshwater Health Index^32^ approach and the results of Souter et al.’s^33^ Se Kong, Se San, and Sre Pok (3S) River Basins baseline assessment. A complete dataset that covers all the inputs and results discussed in this work to assess future sustainability challenges in their social, hydrological, and ecological dimensions for the 3S River Basin are presented in https://doi.org/10.17605/OSF.IO/K6HV4. In Figure S1, we show the geographic layout of the 3S River Basin within the Mekong River Basin.

### Hydrological Model—Lower Mekong River Basin

A compilation^53^ of daily streamflow time series data at nine gauges located at five different countries in the Mekong region (Thailand, Laos People׳s Democratic Republic (PDR), Myanmar, Cambodia, and Vietnam), a processed satellite-based daily precipitation and air temperature (minimum & maximum) data, digital elevation model, refined land cover land use raster data that contains 18 classes that cover agriculture, urban, range and forests land cover land use classes, and tabulated soil data that contains physical and chemical characteristics needed by physically based hydrological models to simulate the cycling of water flux in the Mekong Basin have been used for this work^30,40^. We have presented a physically-based hydrologic model (i.e., the Soil and Water Assessment Tool^54^) for the Lower Mekong River Basin^40^ that ingests both ground-based and satellite-based earth observation data. Our Lower Mekong River Basin hydrological model is properly configured to address common data problems experienced in transboundary basins like the Mekong River (e.g., inconsistency, scarcity, poor spatial representation, difficult access, incompleteness of the available in situ data … etc.). The Lower Mekong River Basin hydrological model^40^ has been calibrated and verified with daily and monthly streamflow data at different parts of the Lower Mekong region^30,40^. For this work, we developed a scenario experiment using climate change and dam data discussed below to examine their future impacts on water resources at the 3S River Basin.

#### Climate data

The NASAaccess tool^52^ which is designed to provide water management tools to those most in need of water security around the world have been utilized for this work. A seamless ingestion of climate change data obtained from the NASA Earth Exchange Global Daily Downscaled Projections (NEX-GDDP)^31^ has been done for our efforts to examine the future freshwater sustainability at the study area. The NEX-GDDP dataset is comprised of downscaled climate scenarios for the globe that are derived from the General Circulation Model GCM runs conducted under the Coupled Model Intercomparison Project Phase 5 CMIP5^36^ and across two of the four greenhouse gas emissions scenarios (RCP 4.5, RCP 8.5) known as Representative Concentration Pathways RCPs^35^. The CMIP5 GCM runs were developed in support of the Fifth Assessment Report of the Intergovernmental Panel on Climate Change IPCC AR5. This dataset includes downscaled projections from the 21 models and scenarios for which daily scenarios were produced and distributed under CMIP5. Each of the climate projections includes daily maximum temperature, minimum temperature, and precipitation for the periods from 1950 through 2100. The Bias-Correction Spatial Disaggregation BCSD method used in generating the NEX-GDDP dataset is a statistical downscaling algorithm specifically developed to address the current limitations of the global GCM outputs^31,55–57^. The NEX-GDPP climate projections are downscaled at a spatial resolution of 0.25 degrees. Future simulations of water flux for the Lower Mekong River Basin were obtained by driving the Lower Mekong River Basin hydrological model^40^ with the downscaled climate data with a spatial grid points of 0.1 degrees following nearest point methods^40^.

The Coupled Model Intercomparison Project Phase 5 (CMIP) groups studied for this work are outlined in Table S1. The selected climate groups used for this work were obtained from previous works^24,34,58^ that discussed recommended climate groups for the Lower Mekong River Basin. The climate groups data used for this work has been adjusted by correction factors obtained by comparing the CMIP5 projection ensembles hindcast data with observed precipitation from the Integrated Multi-satellite Retrieval for the Global Precipitation Measurement mission (GPM-IMERG) remote sensing data products^59^. The suitability of the GPM-IMERG data product to conduct hydrological modeling for the Mekong study area has been previously discussed by Mohammed et al.^40^. The inconsistency, scarcity, poor spatial representation, as well as difficult access and incompleteness of the available in-situ precipitation data have forced us to adopt the use of the GPM-IMERG data product as ‘proxy reality’. Mohammed et al.^40^ found that precipitation forcing data from GPM-IMERG tend to be more skewed in the northern part of the Lower Mekong River Basin in comparison with the southern part. To assess the sensitivity of the GPM-IMERG in hydrological modeling, Mohammed et al.^40^, found that adjusted GPM-IMERG data products tend to overestimate simulated discharge by about 13% in general. Figure S2 gives the CMIP5 projection ensembles hindcast data and how it compares to GPM-IMERG precipitation over the Lower Mekong. Figure S3 gives the CMIP5 precipitation and air temperature projection under the greenhouse gas emissions scenarios (RCP 4.5, RCP 8.5) over the Lower Mekong region. We note an annual trend of about + 6 mm/year across the climate models studied. The climate projections data confirm a change in wet season precipitation patterns, with shorter rainy seasons but higher intensity^24,34,58^. Regarding air temperature projections, the representative concentration scenario (RCP 8.5) climate data suggests that the mean annual maximum and minimum air temperature over the Lower Mekong is expected to increase by an upper maximum limit of about 4.4 °C (277.55 K) and a lower minimum limit of about 3.2 °C (276.35 K) during the 2024–2099 time period. The mean annual minimum air temperature over the Lower Mekong under the RCP 8.5 scenario is expected to increase between 2.7 °C and 4.4 °C (275.85 K and 277.55 K) during the 2025–2099 time period (Figure S3).

#### Dams data

Georeferenced data for existing and proposed dams within the Se Kong, Se San, and Sre Pok (3S) River Basins that contains reservoir area and storage used for this work was obtained from the Greater Mekong Consultative Group for International Agricultural Research (CGIAR) Program on Water, Land and Ecosystems^60^, the Mekong Dam Monitor^61^, the Mekong River Commission^62^, the Food and Agriculture Organization of the United Nations^63^, in addition to personal communications with multiple stakeholders in the 3S region (Table S2). For this work, we examined two dry season reservoir rules (i.e., hypothetical) in addition to the current ones to examine the tradeoffs across human and environment needs for future freshwater sustainability. The three dry season reservoir release rule scenarios used for this work are: (a) Business as Usual (BAU), which follows the current rules (b) Storage, which is a 50% reduction in dry season releases and aims to determine the impact of storing water, and (c) Release, which is a 100% increase in dry season water release depicting increased demand for power in the dry season. The dry season discharges for the various reservoirs modeled are described in Table S2. The wet season reservoir rules are specified as (a) when the reservoir water volume exceeds the maximum reservoir volume, all water in excess of the maximum reservoir volume is released plus the water volume corresponding to the release rules specified in the dry season or the incoming flow (whatever is greater), (b) when the reservoir water volume exceeds the operational reservoir volume but less than the maximum reservoir volume, all water in excess of the operational reservoir volume is released following dry season rules or incoming flow (whatever is greater). The 3S River Basin flows are usually very high during June, July, August, and September compared with other flows during other months^30^.

### Freshwater Health Index

The Freshwater Health Index^32^ (FHI) is a social-ecological assessment framework that assesses three components of freshwater health: Ecosystem Vitality, freshwater ecosystem condition; Ecosystem Services, water-associated provisioning, regulating and cultural services; and Stakeholders & Governance, those who have an interest in, or influence over, freshwater ecosystems and the rules, regulations and institutions by which they are governed. The FHI and its indicators are oriented toward management and stakeholder engagement, and they provide a systematic, quantitative tool that supports the integration between social and ecological nature of freshwater at the basin level. We selected sub-indicators from each of the three FHI components: two indicators of Deviation from the Natural Flow Regime (DvNF) and Base Flow Index (BFI) as indicators of Ecosystem Vitality; Flood Regulation as an indicator of a regulating Ecosystem Service and the full suite of Governance and Stakeholders indicators.

#### Deviation from Natural Flow—DvNF

In stream/river dominated systems, the deviation from natural flow (DvNF) was captured using the Amended Annual Proportion of Flow Deviation index^39^:

1 $$AAPFD = { }\mathop \sum \limits_{j = 1}^{p} \frac{{\sqrt[2]{{\mathop \sum \nolimits_{i = 1}^{12} \left[ {\frac{{m_{i} - n_{i} }}{{\underline{{n_{i} }} }}} \right]^{2} }}}}{p}$$

where $m_{i}$ is monthly flow data accruing to current condition, $n_{i}$ is modeled natural flow for the same period, $p$ is the number of years, and $\underline{{n_{i} }}$ is mean reference flow for month $i$ across $p$ years. The non-dimensional index ($DvNF$) values used for this work are normalized to a 0–100 scale using thresholds reported as follows:

2 $$DvNF = \left\{ {\begin{array}{*{20}l} {100 - 100 \times AAPFD} \hfill & {{\text{for}} \;0 \le AAPFD < 0.3} \hfill \\ {85 - 50 \times AAPFD} \hfill & {{\text{for}}\; 0.3 \le AAPFD < 0.5} \hfill \\ {80 - 20 \times AAPFD} \hfill & {{\text{for}} \;0.5 \le AAPFD < 2} \hfill \\ {50 - 10 \times AAPFD} \hfill & {{\text{for}}\; 2 \le AAPFD < 5} \hfill \\ 0 \hfill & {{\text{for}} \;AAPFD \ge 5.} \hfill \\ \end{array} } \right.{ }$$

The vitality scores for the 3S Rivers results under different management scenarios envisioned are presented in Figure S4. The 3S Rivers in Figure S4 are depicted with a color scale, where red color river segments refer to river segments with anticipated high deviation from natural flow (i.e., DvNF = 50 to 60) and blue river segments refer to low deviation from natural flow river segments (i.e., pristine rivers). The DvNF results shown in Figure S4 are calculated for the time period during 2025 to 2050.

#### Base Flow Index—BFI

The Base Flow Index (BFI)^41^ is the ratio of the annual lowest daily flow to the average daily flow multiplied by 100 during a calendar or water year. The BFI is one of the flow variables thought to influence ecological processes in rivers since it indexes the flow stability. Low flow disturbance is a streamflow classification commonly studied to assess healthy stream ecosystems^64,65^.

#### The Flood Regulation Indicator

This indicator converts the flood duration information into a scale of 0 to 100 where 0 indicates a low capacity in the basin to regulate floods and thus, increased risk of flooding. The flood duration used in this work refers to the number of days per month when reservoir storage volume equals or exceeds 95% of the maximum reservoir storage volume. The maximum reservoir storage volume for each reservoir used in this work is given in Table S2 as storage capacity Full Supply Level (FSL). The projected daily reservoir storage volumes for each reservoir were obtained from Mohammed et al.^30,40^.

To calculate the indicator, storage volume time series for each reservoir is examined. ‘Failure’ in regulating flood in this case is when reservoir storage volume equals or exceeds 95% of the maximum reservoir storage volume. During the time period studied for this work, i.e., 2025–2050, for each month reservoir volume is checked to measure the number of days volume exceeds this threshold. A frequency table is then constructed with a number of columns representing *SUs* and a number of rows representing period intervals. We set the interval period for this work to be 5 years, so our frequency table had 5 rows and 23 columns. We then calculated the ‘Scope’ (i.e., the number of reservoirs with flood regulation issues) and ‘Frequency’ (i.e., number of times with flood regulation issues) to drive the flood regulation capacity of the ecosystem as follows:

(a) ‘Scope’ is calculated as:

3 $$F_{1} = \left( {\frac{No.\, of\, SU\, failed}{{Total\, number\, of\, SU}}} \right) \times { }100.$$

(b) ‘Frequency’ is calculated as:

4 $$F_{2} = \left( {\frac{Number\; of\; instances \;failed}{{Total \;number \;of\; instances }}} \right){ } \times { }100.$$

Then, the score is calculated as:

5 $$Flood\; Regulation\; Capacity = 100 - \sqrt {F_{1} \times F_{2} } \;\left( {{\text{Medium }}\;{\text{evidence}}} \right).$$

#### Governance and Stakeholder Survey

Souter et al.^33^ implemented the FHI Governance & Stakeholders questionnaire survey which assessed the views of 26 representative stakeholders (from each of the three riparian nations plus two representatives of regional international organizations) with knowledge of the 3S’s governance system. The Governance & Stakeholders survey comprises four major indicators—Enabling Environment, Stakeholder Engagement, Effectiveness, and Vision and Adaptive Governance—within which are 12 sub-indicators. Fifty-one questions were asked, each using a 1–5 Likert-type scale to quantify the responses. Questions were phrased so that higher scores corresponded to a more positive assessment. Full details of the governance survey details are given in Table 2. Whilst Souter et al.^33^ provided summary results we present in the supplementary information the results of seven indicators for more nuanced assessment (Figure S6–Figure S10).

Supplementary Information accompanies this paper.

## Supplementary Information

Supplementary Information.

## Data Availability

The data that support the findings of this study are available from https://doi.org/10.17605/OSF.IO/K6HV4.

## Code availability

The code that supports the findings of this study is available from the corresponding author on request.
