# Supplementary material for: Diagnosing challenges and setting priorities for sustainable water resource management under climate change
Source: Sci Rep. 2022 Jan 17;12:796. doi: 10.1038/s41598-022-04766-2 (PMC8764062; doi:10.1038/s41598-022-04766-2)
Supplement: Supplementary file 1 — Supplementary Information. [file 41598_2022_4766_MOESM1_ESM.pdf]

Supplementary Information

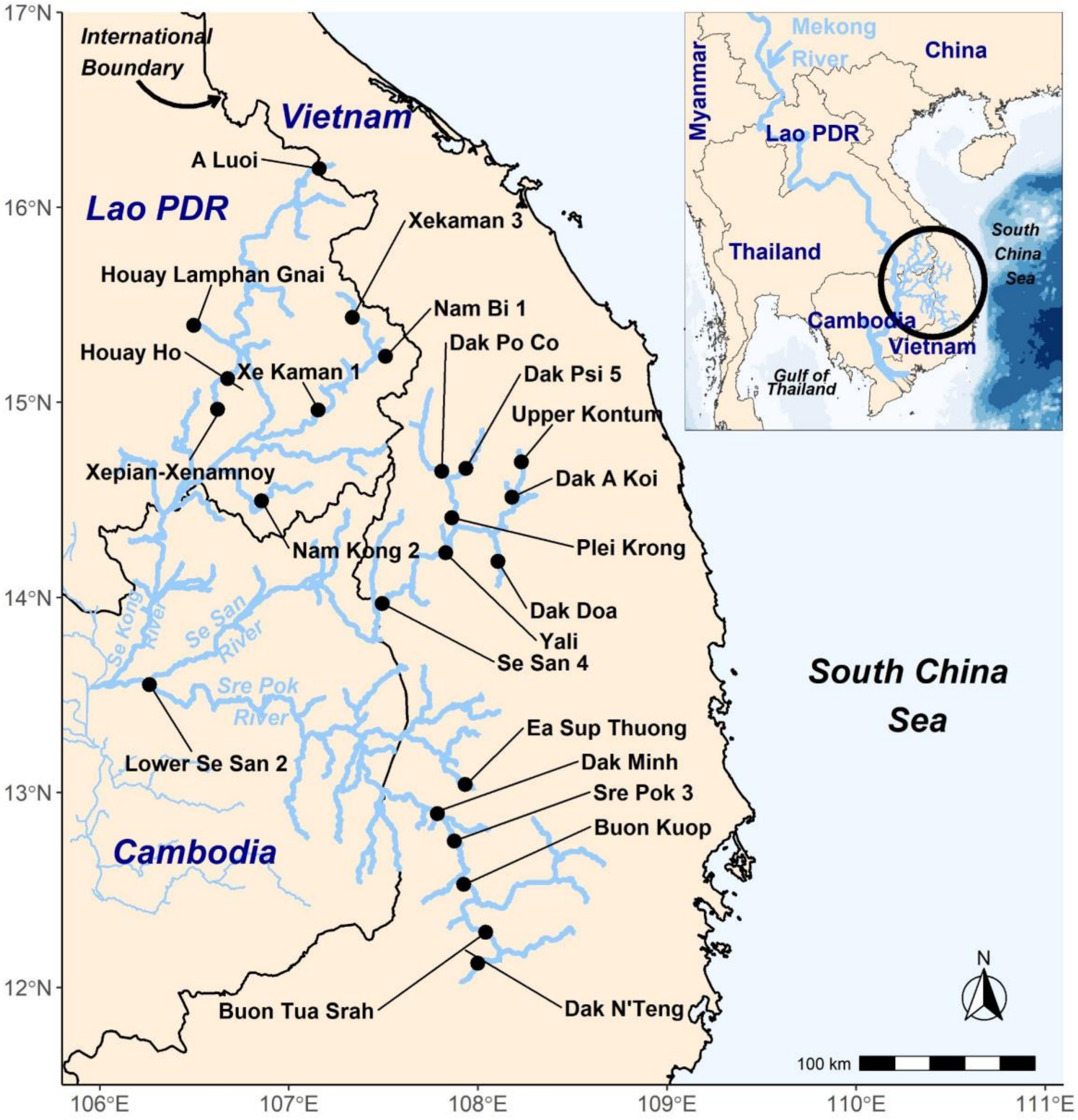

Figure S1. Modeled hydropower dams in the Se Kong Se San, and Sre Pok (3S) River Basin within the Mekong River Basin. The upper right map gives the location of the 3S River Basin within the Mekong River Basin. Black dots are existing and planned reservoirs examined under Business as Usual (BAU), Storage, and Release management scenarios. The 3S River Basin drainage area is

about 78,714 km^2. Maps created and drafted using R: A language and environment for statistical computing version 4.0.3: https://www.R-project.org/ (Vienna, Austria). The map layouts were plotted using EPSG Geodetic Parameter Dataset 4326 projection (https://epsg.io/4326).

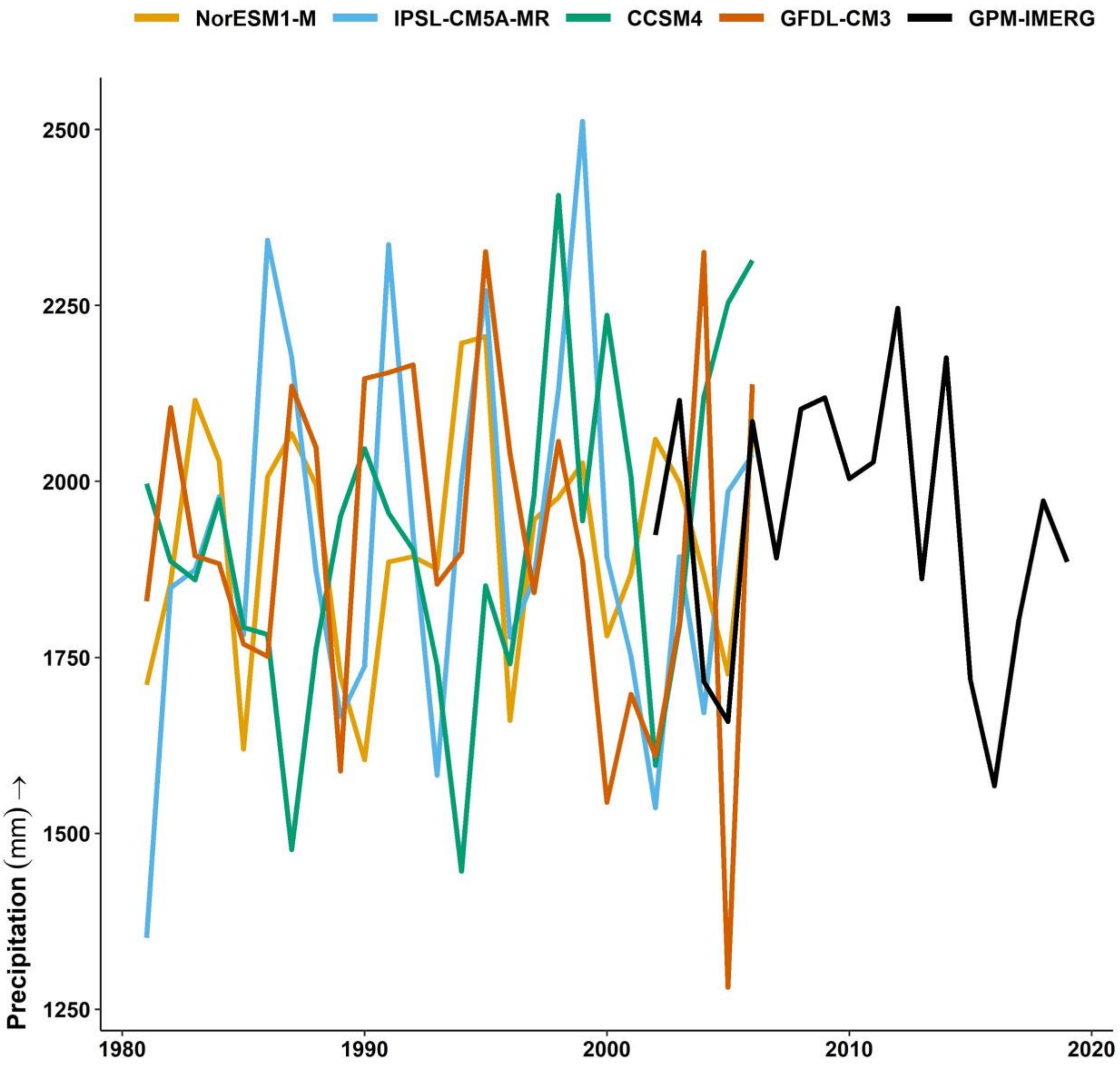

Figure S2. Annual precipitation from the Global Precipitation Measurement mission (GPM) over the Lower Mekong in comparison with CMIP5 hindcast data. The Integrated Multi-satellite Retrieval for the Global Precipitation Measurement mission (IMERG) dataset processed is (GPM_3IMERGDF) obtained from (https://pmm.nasa.gov/data-access/downloads/gpm).

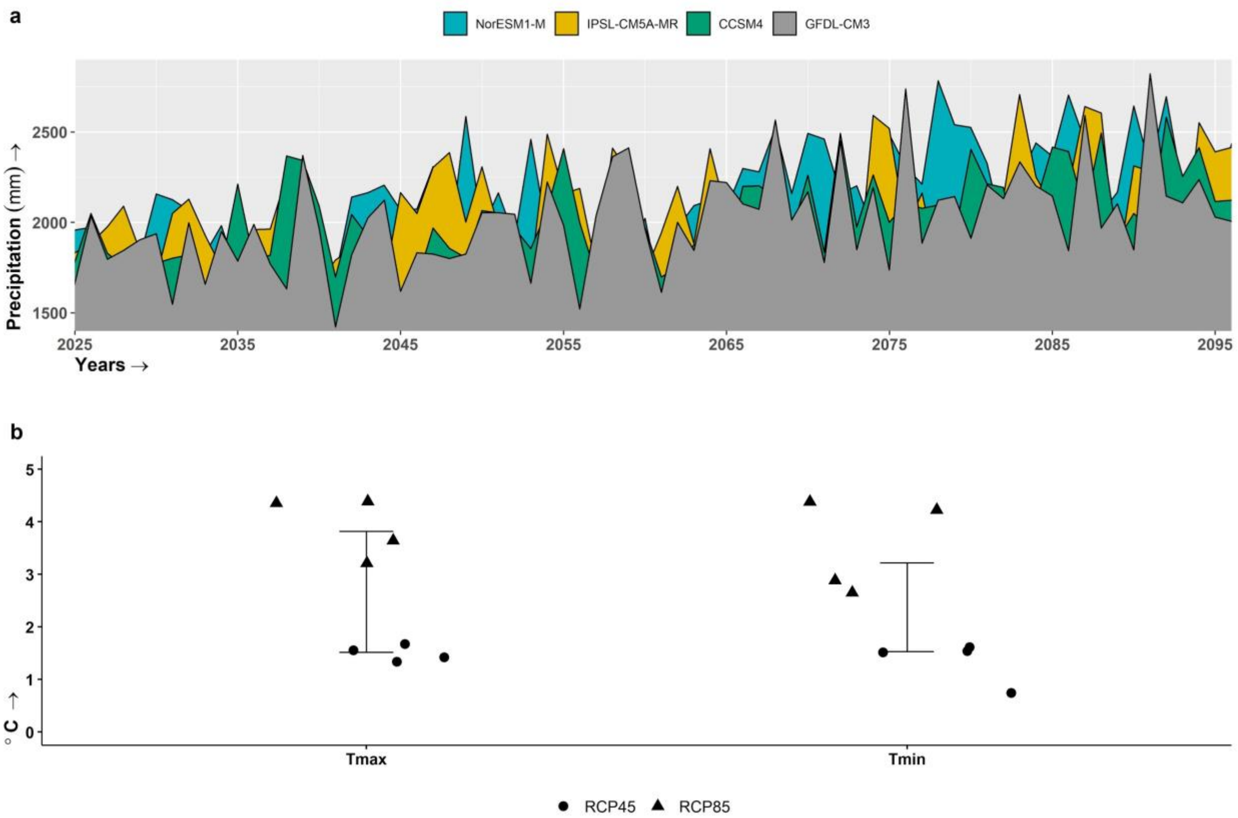

Figure S3. The Lower Mekong River Basin climate projection. Panel (a) gives the projected annual precipitation amounts under the Coupled Model Intercomparison Project Phase 5 (CMIP5) representative concentration scenario (RCP 8.5). Panel (b) displays the projected increase of mean annual air temperatures (Tmin & Tmax). Climate analysis for precipitation and air temperature presented covers the time period from 2024 to 2095. Four climate model groups (NorESM1-M, IPSL-CM5A, GFDL-CM3, and CCSM4) are studied. For air temperature unit conversion, 0 degree Celsius is equal to 273.15 Kelvin.

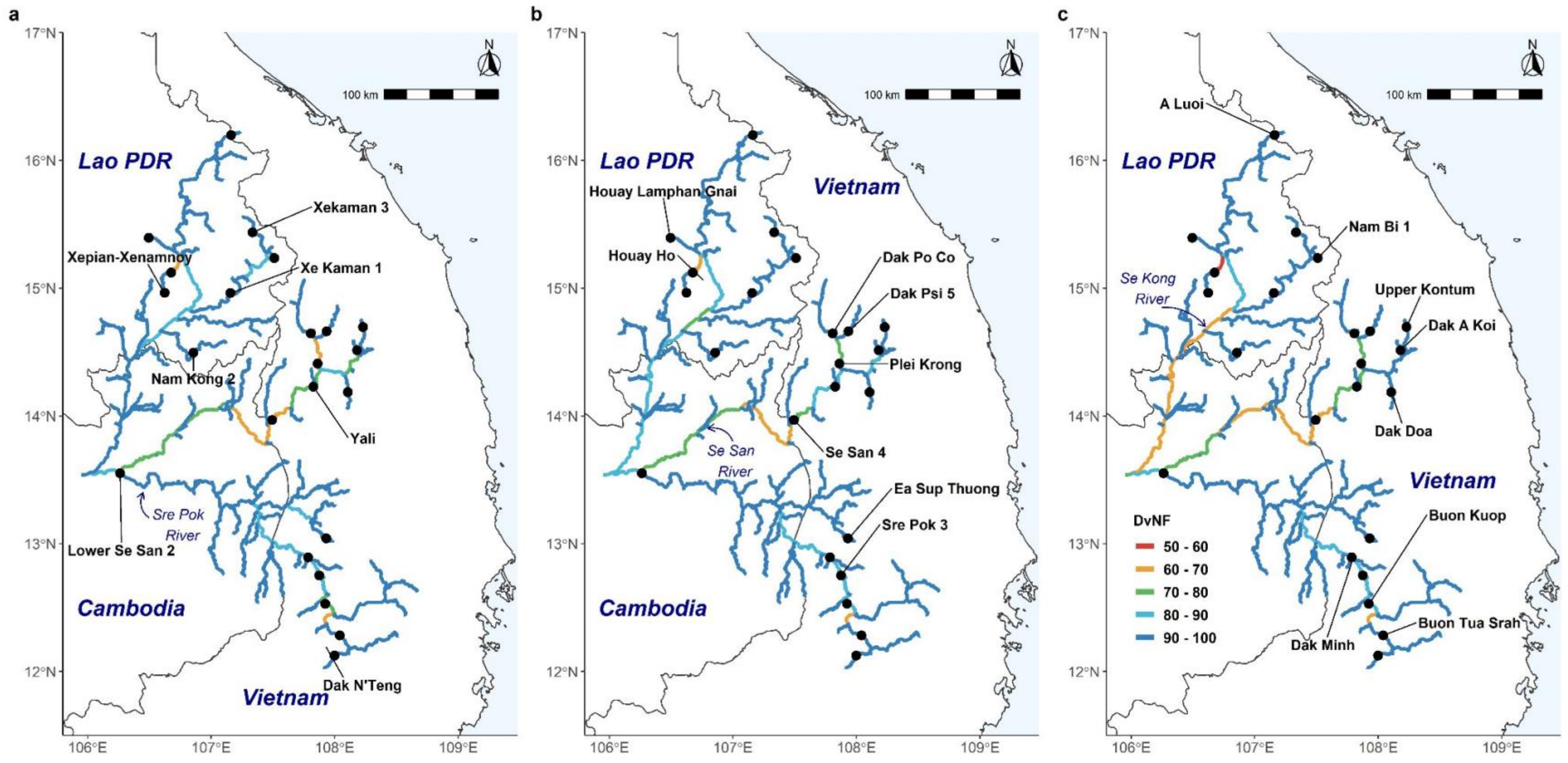

Figure S4. Spatial variation of the deviation from natural flow (DvNF) at the 3S River Basin under the Coupled Model Intercomparison Project Phase 5 (CMIP5) representative concentration scenario (RCP 8.5) with the GFDL-CM3 climate group is displayed for different management scenarios — i.e., panel a (Storage), panel b (Business as Usual, BAU), panel c (Release). Black dots refer to existing and planned reservoirs modeled in the 3S region. Maps created and drafted using R: A language and environment for statistical

computing version 4.0.3: https://www.R-project.org/ (Vienna, Austria). The map layouts were plotted using EPSG Geodetic Parameter Dataset 4326 projection (https://epsg.io/4326).

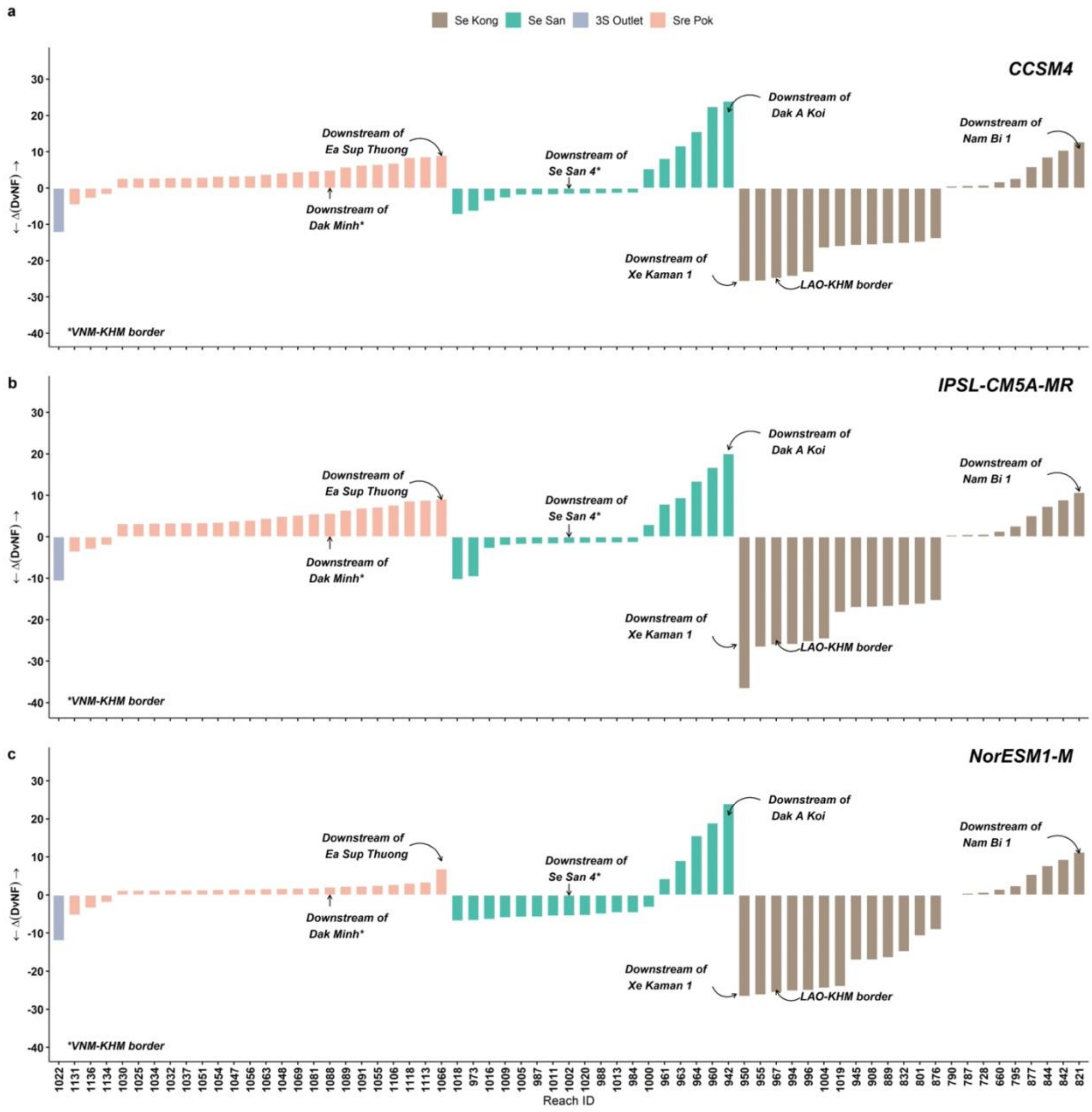

Figure S5. Bar plot of the change in deviation from natural flow ($\Delta\mathrm{DvNF} = \mathrm{DvNF}_{\mathrm{Storage}} - \mathrm{DvNF}_{\mathrm{Release}}$) at the 3S River Basin under the Coupled Model Intercomparison Project Phase 5 (CMIP5) representative concentration scenario (RCP 8.5) with the (a) CCSM4, (b) IPSL-CM5A-MR, and (c) NorESM1-M climate groups. A zero in ΔDvNF refers to 3S River segments that are insensitive to management scenarios. The DvNF results shown were calculated from 2025 to

2050 time period. The 3S River segments are labeled with Reach ID numbers (e.g., Reach ID # 1022 is the 3S Outlet).

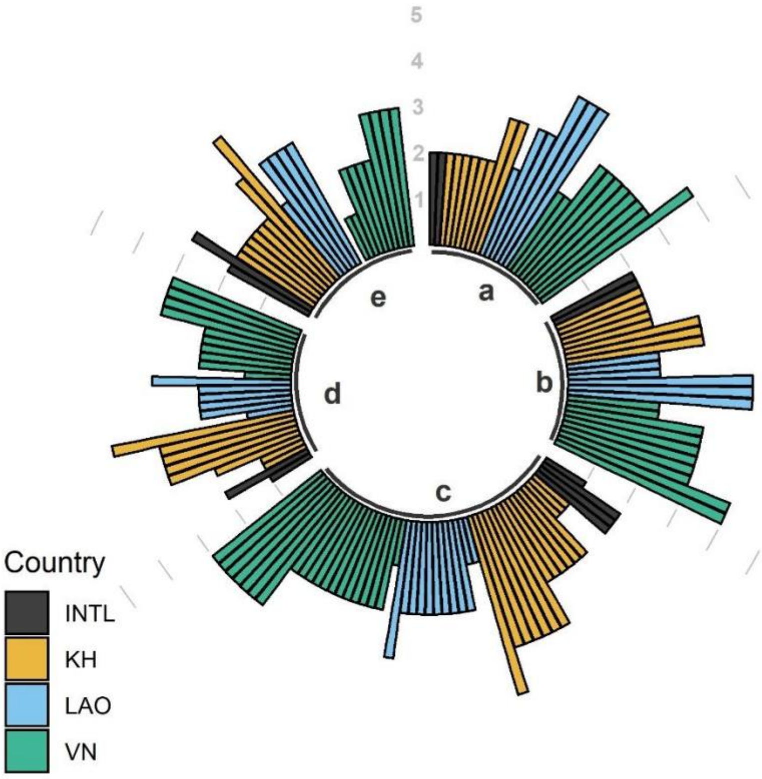

Figure S6. Financial capacity questionnaire responses from the 3S Governance and Stakeholders survey a) level of investment in water supply development, b) level of investment in service delivery systems, c) level of investment in wastewater handling and treatment, d) level of investment in ecosystem conservation and rehabilitation, and e) level of investment in monitoring and enforcement.

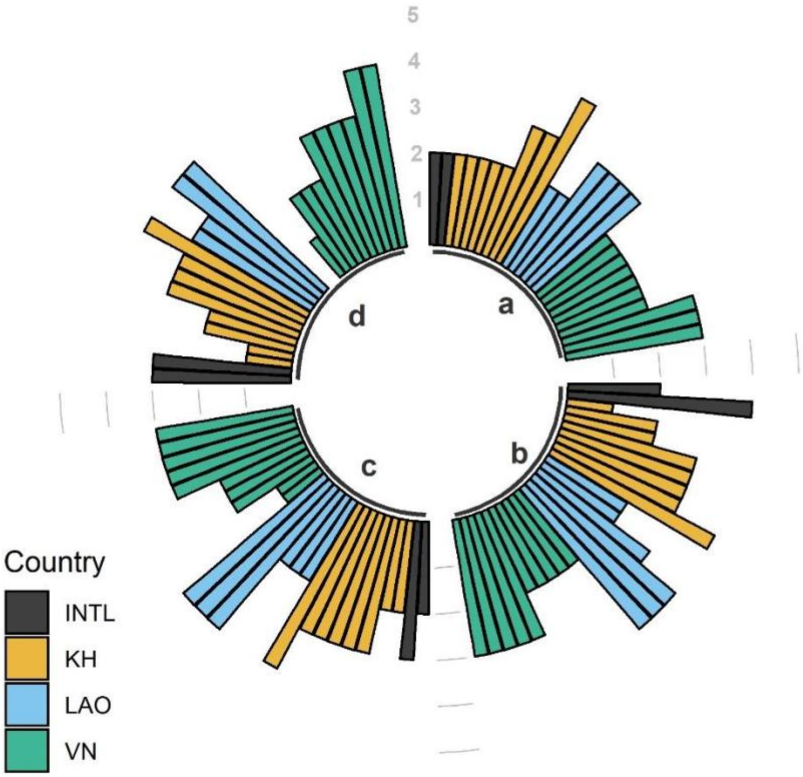

Figure S7. Information access and knowledge questionnaire responses from the 3S Governance and Stakeholders survey. a) information is accessible to interested stakeholders; b) information meets expected quality standards, in terms of frequency, level of detail, and subjects of interest to stakeholders; c) information is transparently sourced; and d) all available, sound and relevant information is routinely applied in decision-making.

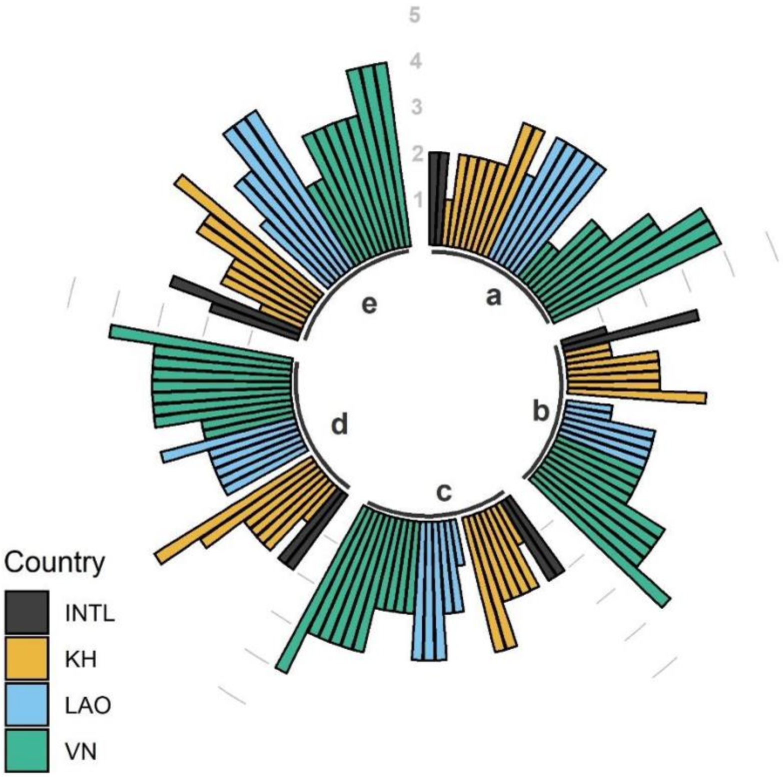

Figure S8. Enforcement and compliance questionnaire responses from the 3S Governance and Stakeholders survey. a) surface water abstraction guidelines are enforced; b) groundwater abstraction guidelines are enforced; c) flow requirement guidelines are enforced; d) water quality guidelines are enforced; and e) land use guidelines are enforced.

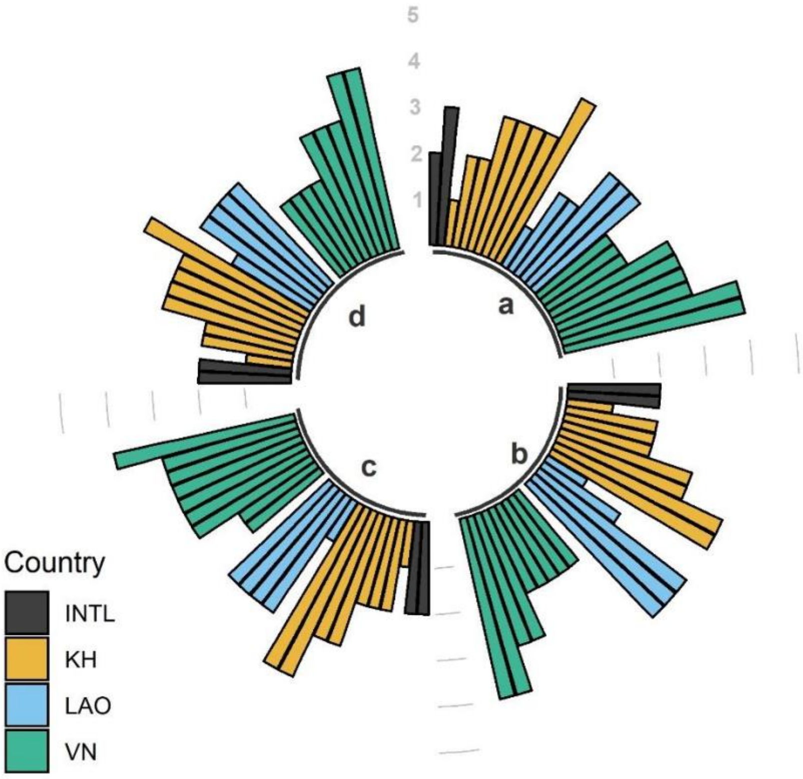

Figure S9. Distribution of benefits from ecosystem services questionnaire responses on the from the 3S Governance and Stakeholders survey. a) economically vulnerable populations benefit from ecosystem services; b) indigenous people benefit from ecosystem services; c) women and girls benefit from ecosystem services; and d) resource-dependent communities benefit from ecosystem services.

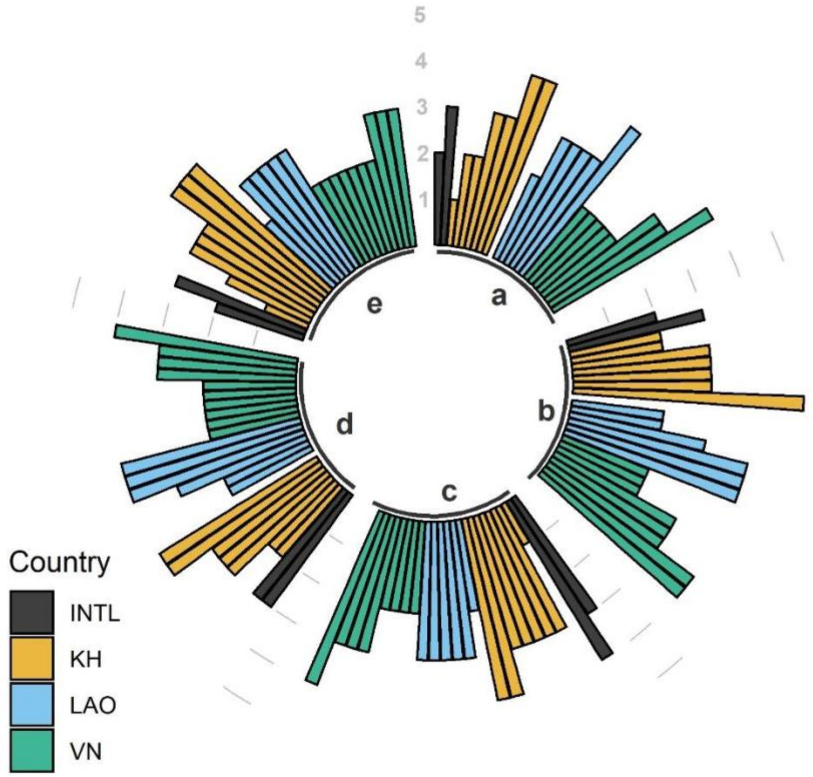

Figure S10. Water-related conflict questionnaire responses from the 3S Governance and Stakeholders survey a) frequency of conflict due to overlapping jurisdictions (e.g., between national governments in transboundary systems, provincial and national government, or between agencies); b) frequency of conflict about water rights allocation; c) frequency of conflict about access; d) frequency of conflict regarding the siting of infrastructure; and e) frequency of conflict over water quality and other downstream negative impacts.

Table S1. The Coupled Model Intercomparison Project Phase 5 (CMIP5) groups examined.

No.	Modeling Center	Institute ID	Model Name
1	National Center for Atmospheric Research	NCAR	CCSM4
2	NOAA Geophysical Fluid Dynamics Laboratory	NOAA GFDL	GFDL-CM3
3	Institut Pierre-Simon Laplace	IPSL	IPSL-CM5A-MR
4	Norwegian Climate Centre	NCC	NorESM1-M

71 Table S2. Current and under development reservoirs used at the Se Kong, Se San, and Sre Pok (3S) River Basins modeling framework.

No	Name	River	Latitude	Longitude	Country	COD	Storage		Dry Season Discharge			Installed Capacity
							Capacity – FSL	Reservoir Area	BAU	Storage	Release	
							(Mm³)	(km²)	(m³/s)	(m³/s)	(m³/s)	(MW)
1	A Luoi	A Sap	16° 11' 51"	107° 9' 43"	Socialist Republic of Vietnam	2012	60.20	20.80	49	24.5	98	170
2	Buôn Kốp	Sre Pok	12° 31' 30"	107° 55' 33"	Socialist Republic of Vietnam	2009	63.24	5.57	100	50	100	280
3	Buon Tua Srah	Sre Pok/Krong Kno	12° 16' 56"	108° 2' 29"	Socialist Republic of Vietnam	2009	786.90	37.10	100	50	100	86

4	Dak A Koi	Dak A Koi	14° 30' 47"	108° 10' 55"	Socialist Republic of Vietnam	2007	150.00	7.00	18	9	36	60
5	Dak Doa	Ia Krom	14° 11' 5"	108° 6' 24"	Socialist Republic of Vietnam	2010	29.13	2.28	35	17.5	70	14
6	Dak Minh	Dak Man	12° 54' 33"	107° 48' 17"	Socialist Republic of Vietnam	N/A	0.15	10.00	195	97.5	195	N/A
7	Dak N'Teng	Dak N'Teng	12° 11' 46"	107° 55' 36"	Socialist Republic of Vietnam	2011	25.49	323.00	5.6	2.8	11.2	13
8	Dak Po Co	Krong Po Ko/Sesan	14° 38' 43"	107° 48' 30"	Socialist Republic of Vietnam	2015	3.39	0.74	83	41.5	166	15
9	Dak Psi 5	Dak Psi	14° 39' 41"	107° 56' 12"	Socialist Republic of Vietnam	2012	2.40	638.00	46	23	92	10

10	Ea Sup Thuong	Ea Sup	13° 2' 4"	107° 55' 56"	Socialist Republic of Vietnam	2004	146.00	15.00	29	14.5	58	—
11	Houay Ho	Houayho, Xekong	15° 3' 34"	106° 45' 52"	Lao People's Democratic Republic	1999	3,530.00	37.00	11	5.5	22	152.1
12	Houay Lamphan Gnai	Xe Kong	15° 21' 36"	106° 29' 54"	Lao People's Democratic Republic	2015	140.00	9.00	20	10	40	88
13	Lower Se San 2	Se San	13° 33' 5"	106° 15' 50"	Kingdom of Cambodia	2019	1,790.00	335.00	195	97.5	195	480
14	Nam Bi 1	Nam Kai	15° 14' 8"	107° 30' 57"	Lao People's Democratic Republic	2021[‡]	3.00	0.03	2	1	4	50

15	Nam Kong 2	Nam Kong	14° 29' 41"	106° 51' 24"	Lao People's Democratic Republic	2021‡	71.40	4.20	55	27.5	110	66
16	Plei Krong	Se San/ Kroong Po Ko	14° 24' 30"	107° 51' 47"	Socialist Republic of Vietnam	2008	1,048.70	53.28	20	10	40	100
17	Se San 4	Se San	13° 58' 6"	107° 29' 43"	Socialist Republic of Vietnam	2009	893.30	58.41	195	97.5	195	360
18	Sre Pok 3	Sre Pok	12° 45' 8"	107° 52' 36"	Socialist Republic of Vietnam	2009	219.00	17.68	130	65	130	220
19	Upper Kontum	Se San/ Dak Bla/ Dak Nghe	14° 41' 39"	108° 13' 48"	Socialist Republic of Vietnam	2011	174.00	7.08	55	27.5	110	250
20	Xe Kaman 1	Xe Kaman	14° 57' 39"	107° 9' 23"	Lao People's Democratic Republic	2015	4,804.00	149.80	55	27.5	110	290

21	Xe Kaman 3	Houayho, Xekong	15° 26' 10"	107° 20' 12"	Lao People's Democratic Republic	2014	141.50	5.20	55	27.5	110	250
22	Xepian-Xenamnoy	Xepian/Xenamnoy	14° 56' 47"	106° 37' 39"	Lao People's Democratic Republic	2018	1,092.00	50.00	6.7	3.35	13.4	410
23	Yali	Se San	14° 13' 39"	107° 49' 47"	Socialist Republic of Vietnam	2001	1,037.00	64.50	195	97.5	195	720

‡: Planned
COD: Commercial Operation Date (i.e., when the dam was commissioned)
BAU: Business as usual for dry season reservoir release discharge
N/A: Not Available
